# Harnessing the Therapeutic Potential of Capsaicin and Its Analogues in Pain and Other Diseases

**DOI:** 10.3390/molecules21080966

**Published:** 2016-07-23

**Authors:** Shaherin Basith, Minghua Cui, Sunhye Hong, Sun Choi

**Affiliations:** National Leading Research Laboratory (NLRL) of Molecular Modeling & Drug Design, College of Pharmacy and Graduate School of Pharmaceutical Sciences, Ewha Womans University, Seoul 03760, Korea; shaherinb@gmail.com (S.B.); babana1987@naver.com (M.C.); sunhye_15@naver.com (S.H.)

**Keywords:** capsaicin, therapeutic, analogues, neuropathic pain, desensitization

## Abstract

Capsaicin is the most predominant and naturally occurring alkamide found in *Capsicum* fruits. Since its discovery in the 19th century, the therapeutic roles of capsaicin have been well characterized. The potential applications of capsaicin range from food flavorings to therapeutics. Indeed, capsaicin and few of its analogues have featured in clinical research covered by more than a thousand patents. Previous records suggest pleiotropic pharmacological activities of capsaicin such as an analgesic, anti-obesity, anti-pruritic, anti-inflammatory, anti-apoptotic, anti-cancer, anti-oxidant, and neuro-protective functions. Moreover, emerging data indicate its clinical significance in treating vascular-related diseases, metabolic syndrome, and gastro-protective effects. The dearth of potent drugs for management of such disorders necessitates the urge for further research into the pharmacological aspects of capsaicin. This review summarizes the historical background, source, structure and analogues of capsaicin, and capsaicin-triggered TRPV1 signaling and desensitization processes. In particular, we will focus on the therapeutic roles of capsaicin and its analogues in both normal and pathophysiological conditions.

## 1. Introduction and Historical Viewpoint

The transfer of foods between the New and Old Worlds during the Columbian Exchange introduced the chili pepper to the world [1]. The consumption of chili peppers by inhabitants of the Tehuacan valley (Mexico) dates back over 8000 years (to between 7000–6000 BC). Archeologists have estimated the cultivation of chili peppers began around 5200–3400 BC, thus categorizing it as one of the oldest domesticated plants [2]. After the discovery of the New World, this small fruit was introduced to Europe and its cultivation started migrating slowly to other parts of the globe. Presently, *Capsicum* is considered as one of the global foods that are found in nearly every country and adopted by different cultures as an essential ingredient in their cuisines. Due to its worldwide consumption, it is recognized under several names including aji, cayenne, red pepper, chili pepper, paprika, jalapeno, and tabasco [3].

The principal component responsible for the piquancy of chili peppers is capsaicin. This naturally occurring irritant was first isolated in 1816 by Christian Friedrich Bucholz in impure form and named it ‘capsicin’ [4]. Later, in 1876, Thresh extracted this compound in almost pure form and renamed it as ‘capsaicin’ [5]. The pure form of the compound was isolated by Micko in 1898 [6]. Nelson first solved its empirical formula and offered a partial chemical structure in 1919 [7]. The original synthesis of the compound was reported in 1930 by Spath and Darling [8]. Since then, several compounds related to capsaicin grouped as capsaicinoids have been isolated from *Capsicum* species [9]. These compounds are believed to be synthesized by the plant as defense substances against the attacks of fungi, microbes, and herbivores [10]. 

Since 7000 BC, *Capsicum* has a long and convoluted history in culinary and traditional medicine [11]. Besides culinary usage, capsaicin has been utilized in a wide array of other applications including insect deterrence, self-defense products (pepper spray), protecting crops from roving herds of elephants (chili-dung bombs), repelling squirrels (capsaicin-flavored bird seeds), self-protectant lotions to ward off sharks, chemical weapons, ornamental plants, and a plethora of medicinal applications [12]. In traditional medicine, *Capsicum* was consumed by people in tropical countries to handle the hot weather by increasing the regulation of heat loss with capsaicin-induced skin vasodilation and perspiration [13]. Other folk medicinal applications include treatments of cough, sore throat, tonsillitis, gastric ulcers, backache, cholera, gout, water retention, rheumatism, cramps, diarrhoea, dyspepsia and toothache, appetite stimulation, and hair growth restoration [14,15]. Despite its widespread utility, the biological aspects of capsaicin were not well understood, until the discovery of its dual actions in sensory neurons: an instantaneous, but temporary excitation succeeded by a long-lasting refractory state, generally known as desensitization [16]. In this state, the previously stimulated neurons are no longer sensitive to its previous or other stimuli. This effect has been exploited for its therapeutic potential [17,18]. The restorative effects of capsaicin began to be revealed in the 19th century. Western people primarily utilized capsaicin for treating itching or burning extremities [19]. Buchheim (1873) and Hogyes (1878) were the first to report the burning symptoms produced by capsicol (partially purified capsaicin) upon contact with mucous membranes and increased gastric juice secretion [20], thus showing its first pharmacological effects.

The past few decades have witnessed unprecedented advances in the field of capsaicin research. As an archetypal member of the transient receptor potential (TRP) family, transient receptor potential (cation) channel of the vanilloid receptor family, subtype 1 (TRPV1) was initially identified as the capsaicin receptor [21]. TRPV1 is a tetrameric, non-selective, ligand-gated cation channel that serves as an integrator of various stimuli including vanilloids, voltage, noxious heat, endogenous lipids, protons / cations, and various inflammatory mediators. Of TRPV1’s agonists, capsaicin acts as a highly selective and archetypal exogenous activator with an EC_50_ of approximately 700 nM on this receptor [22,23]. The initial discovery of TRPV1 heralded a renaissance of interest in the therapeutic exploitation of capsaicin [22]. Capsaicin-containing ointments/creams have been in medicinal utility for decades to relieve chronic neuropathic pain disorders. Topical use of capsaicin has been found to be potent in the management of skin disorders, shingles, muscle strain, arthritis-related disorders, diabetic neuropathy, and other pain disorders. Moreover, the approval of the use of capsaicin drugs strengthened its clinical importance. A number of pharmaceutical companies market topical capsaicin under the trade names such as Menthacin, Zostrix, and Capzasin-P [15]. In 2009, the European Union (EU) and Food and Drug Administration (FDA) approved the use of capsaicin 8% patch (Qutenza or NGX-4010) for the treatment of neuropathic pain conditions. EU supported the application of Qutenza for post-herpetic neuralgia (PHN), peripheral neuropathic pain (PNP), and HIV-associated distal sensory polyneuropathy (HIV-DSP) related pain conditions [24,25], however, in the USA, the FDA approved its use for PHN only [26,27]. These reports and several other studies clearly depicted capsaicin as an exciting pharmacological agent and its application in different clinical conditions are still being investigated [28,29]. Apart from its pleiotropic pharmacological and physiological roles, capsaicin has also been studied for its exhibited antimicrobial and antivirulence activities [30], where it is used as a potent natural inhibitor of pathogenic microorganisms in food [31]. Conversely, the use of capsaicin as a culinary spice or drug has been limited due to its increased irritation, unpleasant hot sensation, and nociceptive activity. This has led to the search of its non-pungent analogues without the inherent and unwanted side-effects which will improve the production of more efficient and tolerable drugs. The aim of this review is to provide a comprehensive overview of the capsaicin compound, and more importantly delve into its therapeutic potential in various human ailments. We also briefly discuss the adverse clinical outcomes and future therapeutic directions of capsaicin.

## 2. Source, Structure, and Analogues of Capsaicin

**Figure 1:**
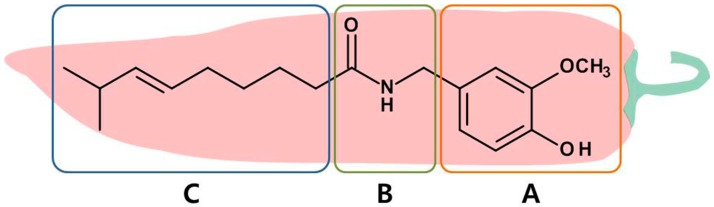
Chemical structure of capsaicin, the primary ingredient of chili pepper, and its three important regions, namely A (aromatic head), B (amide linkage) and C (hydrophobic tail) depicted in different colored boxes.

Capsaicin (*trans*-8-methyl-*N*-vanillyl-6-nonenamide) is a natural vanilloid identified from various chili peppers. It is found in adequate amounts in the placental tissue (which holds the seeds) and to a lesser extent in the seeds and pericarp portions of *Capsicum*. Capsaicin is a highly volatile, hydrophobic, odorless, and colorless alkaloid with a molecular weight of 305.4 kDa and melting point of 62–65 °C. Structurally, capsaicin belongs to a group of chemicals known as vanilloids. The structure of capsaicin consists of a vanillyl (methylcatechol) head group (A-region) and an aliphatic tail (hydrophobic—C-region) linked by a central amide bond (B-region) as depicted in Figure 1. 

This exact combination of these regions is accountable for the pharmacological activities of capsaicin. The pungency of capsaicin is due to the presence of the vanillyl moiety, which is also responsible for its adverse effects in clinical use [16]. Various structure-activity relationship (SAR) studies imply that the vanillyl and amide bond moieties are involved in maintaining the excitation of sensory neurons and the aliphatic tail provides maximal potency [32,33]. The initial burning pain sensation caused by capsaicin was considered as a potential drawback to its use and development as an active principle in formulations for the treatment of pain and itch [16]. In an attempt to search for compounds less pungent than capsaicin, several studies focused on the design and development of more potent capsaicin derivatives with less toxic effects and less or no pungent action.

**Figure 2:**
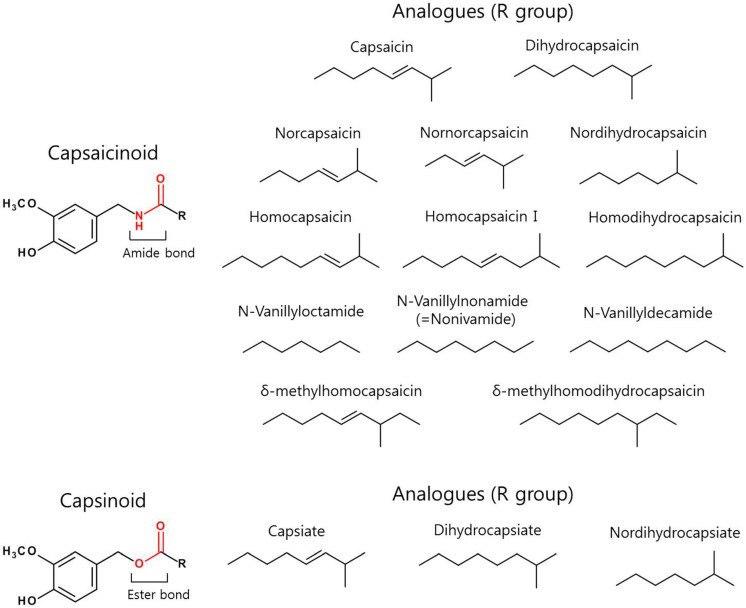
The general chemical structure of capsaicinoid and capsinoid and their related analogues that differ in the R-group are summarized. Capsaicinoids and capsinoids have a common vanillyl moiety and differ in the central linkage (shown in red) and R-group.

Several capsaicin analogues with similar functional groups but possessing variations in one or more of the three structural moieties exist [34,35]. Capsaicin and capsaicinoids which are responsible for the pungent sensation in chili peppers are produced by all plants of the genus *Capsicum*, with the exception of *Capsicum annum* (bell pepper). The actual percentage of capsaicinoids varies depending on the pepper variety and extraction method. The content of capsaicinoids in naturally occurring spices range from 0.1 mg/g in chili pepper to 2.5 mg/g in red pepper and 60 mg/g in oleoresin red pepper [36,37]. The capsaicin content of different peppers determined by liquid chromatography techniques range from 0.1 to 4.25 mg/g of pepper [37]. Other pepper varieties such as *Capsicum frutescens*, *Capsicum annuum*, and *Capsicum chinense* were found to contain 0.22 to 20 mg of total capsaicinoids/g of pepper (dry weight) [38]. The ‘hotness’ of a pepper is measured by the Scoville scale that considers the number of times the chili extract must be diluted to make the pungency imperceptible. The piquancy of different peppers depends on its capsaicin content ranging from 0 Scoville heat units (SHU) in bell pepper to 16 million SHU in pure capsaicin. There are more than twenty capsaicinoids (presented in Figure 2) identified from *Capsicum* species [39]. The most predominant forms present in *Capsicum* are capsaicin and dihydrocapsaicin that account for 80%–90% of the capsaicinoids, whereas others exist in smaller quantities. All capsaicinoids have a similar structure, varying only by the length of aliphatic side chain and degree of saturation (presence or absence of double bonds) in the alkyl side chain region. Capsaicinoids possess potentially valuable pharmacological and physiological properties including analgesic, anti-cancer, anti-obesity, anti-inflammatory, and anti-oxidant. Hence, they may show potent therapeutic value in cancer prevention, cardiovascular and gastrointestinal systems, pain relief, and weight loss [40]. Another group of compounds, namely the capsinoids (naturally occurring in ‘CH-19 Sweet’ peppers) show similar effects like capsaicinoids, but differ slightly in the structure (the central linking group is an ester—see Figure 2), display non-pungent characteristic and are easily broken down in a normal aqueous environment. Capsinoids are found to be less toxic than capsaicinoids and possess anti-cancer, anti-obesity, and anti-oxidant biological properties. Besides TRPV1 activation, capsinoids also stimulate transient receptor potential (cation) channel of the ankyrin-like receptor family, subtype 1 (TRPA1), in a less potent manner [41]. TRPA1 is one of the members of TRP channel family that serves as an irritant for several electrophilic compounds and is predominantly expressed in the TRPV1 positive subset of sensory neurons [42,43]. It has been reported to play a functional role in pain and neurogenic inflammation, since it acts as a target for mustard oil in peripheral sensory neurons. Additionally, it also participates in other sensory functions, including, cold sensation and hearing [44].

## 3. Capsaicin-Induced TRPV1 Signaling, Desensitization and Sensitivity

Researchers have long been unaware of the receptor for capsaicin, though pronociceptive and antinociceptive effects of capsaicin were well established. Upon the advent of cDNA clone encoding capsaicin-stimulated receptor by Julius and colleagues in 1997, the molecular basis of TRPV1 was finally recognized [22]. Since then, there has been much interest in manipulating with the pharmacology of capsaicin and its receptor. TRPV1 is a well-characterized Ca^2+^ permeant polymodal receptor that is activated by several exogenous chemical activators (including vanilloids), protons, and endogenous activators [23]. It acts as a potent heat sensor in peripheral sensory neurons, thus exhibiting a high sensitivity to heat with Q10 values of approximately 25 [22]. In addition, it is also activated by changes in membrane voltage (+60 mV) and at noxious temperatures with thresholds near 43 °C, thus playing a critical role in nociceptor and inflammatory thermal sensation [45]. TRPV1 is expressed at high levels in the small-diameter sensory neurons of the dorsal root, trigeminal, and vagal ganglia [23]. The solved three-dimensional structure of TRPV1 is comprised of four symmetrical subunits, and each subunit consists of four recognizable modules: the voltage sensor and pore modules in the transmembrane (TM) region and the long cytosolic N- and C-terminal modules [46]. TM region has six TM-spanning helices, S1–S6, which assembles to form homo- or hetero-tetramers with neighboring subunits. These four subunits make up the central ion-conducting pore that opens up in response to the agonists. Interestingly, TRPV1 receptor has distinctive dual gates, upper gate (G1) and lower gate (G2) in the channel pore that control current flow. These two gates undergo major structural rearrangements in the activated state of the channel [21]. 

Capsaicin produces burning and itching sensations upon binding to TRPV1 receptor, followed by the activation of polymodal C and Aδ nociceptive fibers [22,47]. However, the binding mode of capsaicin with TRPV1 at the atomic level remained unclear, until the recent release of high-resolution cryo-EM solved agonist-bound TRPV1 structures [48,49]. The molecular details underlying capsaicin binding and activation of TRPV1 have been well documented [48,49]. Recently, the binding site, partitioning behavior, and capsaicin interaction with DMPC lipid bilayer membrane has also been studied using its intrinsic fluorescence [50]. In the agonist-bound state, capsaicin binds to the region that spans TM3 to 4 of the TRPV1 receptor. The bound capsaicin adopts ‘tail-up, head-down’ configurations where the vanillyl and amide groups form specific interactions that anchor the agonist to the receptor. Moreover, the channel’s active state is stabilized by ‘pull-and-contact’ interactions between the capsaicin head region and S4-S5 linker of receptor [49]. Residues of murine TRPV1 including, Y512 (helix S3), S513 (helix S3), E571 (S4–S5 linker), and T671 (helix S6) bind to the ring region of capsaicin through hydrogen-bonding, whereas, T551 (helix S4) binds to the amide region of capsaicin. Furthermore, no changes has been observed in the selectivity filter that is present in the G1; however, G2 is significantly expanded to 7.6 Å [46]. A large shift of approximately 2.7 Å in the S4–S5 linker and an outward movement of I679 have been observed in the capsaicin-bound form. Upon TRPV1 activation by capsaicin, the probability of the channel being in the open state is increased, thereby resulting in the Ca^2+^ influx. When the receptor is activated in periphery, it leads to depolarization of the nerve terminal and generate an action potential which transmits information to the brain through the spinal cord and higher brain regions to somatosensory cortex. Although TRPV1 acts as a thermo-sensitive channel, it is also activated by low pH at the channel extracellular surface. Structurally, the channel has putative proton sites at the extracellular region [51]. Proton sensing extracellular residues, E600 and E648, potentiates two different effects of pH on TRPV1. Particularly, a strongly acidic pH induces channel activation, whereas, basic pH values reduce the channel activation threshold [52,53]. Helix pore residues, T633 and V538, and S3–S4 connecting loop play an important role in the activation of TRPV1 by low pH. These two amino acids direct the gating mechanism through hydrophobic interactions. Additionally, F660 also plays a key function in potentiation and proton stimulation of TRPV1 [21]. It is well-known that the receptor is also activated at higher temperatures. This thermal activation protects the body from harmful heat stimuli by coordinating the withdrawal from the area of contact to prevent any burn injury. TRPV1 stimulation by heat remains obscure, however it is proposed that the C-terminus and outer pore region of the channel play potent roles [54]. Thus, the two processes, namely, sensitization and desensitization, can be regarded as potent built-in biological safety mechanisms that act as a check point for over- activation of the receptor. Besides its irritant effect, a long-term sensory neuron blocking action of capsaicin which can be used in the functional exploration of sensory neurons was discovered. Since then, several studies utilized capsaicin as a potent probe in the investigation of sensory neuron mechanisms [55]. Depending on the dose level and delivery route, capsaicin can selectively activate, desensitize, or exert a neurotoxic effect on sensory neurons.

Binding of capsaicin to TRPV1 opens the ion channel. However, when constantly activated, the receptor becomes desensitized and the pore is no longer permeable to ions. In order to reactivate the receptor, the channel has to go through the closed state before opening again. Capsaicin desensitization differs from sensory adaptation because a delay of few minutes is required for capsaicin desensitization to take place [56]. Two different types of desensitization have been linked to TRPV1 receptors: functional and pharmacological desensitizations [57]. Functional desensitization refers to short-term local application to nerve endings leads to reduction or loss of responsiveness of sensory neurons to other stimuli. The second type of pharmacological desensitization refers to the gradual decrease in the response that is normally evoked by capsaicin, following repeated administrations of that agonist. Both these desensitization processes require extracellular Ca^2+^, suggesting the involvement of Ca^2+^ dependent intracellular signaling mechanisms. At much higher capsaicin concentrations than required to activate TRPV1, mitochondrial impairment has been observed by the direct inhibition of electron transport chain [58]. However, if TRPV1-expressing sensory nerve fibers are exposed to high- or low-concentration of capsaicin for a prolonged period, higher amounts of intracellular Ca^2+^ and other subsequent processes can lead to its impairment for extended periods [59]. Moreover, subsequent release of central neurotransmitters including substance P (SP) and glutamate from nociceptive nerve fibers is also observed. These nociceptive nerve fibers become ‘chemically denervated’ and functionally silent due to the exhaustion of transmitters further leading to the desensitization of the channel [60]. This capsaicin-induced desensitization aspect has been widely used for centuries to control pain. Pharmacological desensitization can be ablated by inhibitors of Ca^2+^-dependent phosphatases, thereby highlighting the importance of phosphorylation in receptor activation [61]. Moreover, protein kinase A (PKA)-mediated phosphorylation is responsible for reversing pharmacological desensitization [62]. The rapidity and extent with which desensitization to capsaicin occurs is related to its dose levels, duration of exposure, and the time interval between consecutive dosings. It has been reported that with low suprathreshold doses of capsaicin administered at appropriate intervals, desensitization does not occur. However, at higher doses of capsaicin and prolonged exposure, desensitization occurs. The duration of the desensitization process may occur varying from a few hours to a few days [63,64]. Besides desensitization, repeated application of capsaicin has also been involved in the degeneration of epidermal nerve fibers in parallel to pain reduction responses [65,66]. This effect is a reversible process, which explains the long-lasting antinociceptive effects of capsaicin. Capsaicin-triggered degeneration of small nerve endings is found to occur through neurotoxicity due to increased Ca^2+^ uptake or intracellular reactive oxygen species (ROS) production [67,68]. Neurotoxicity is partially due to increased osmotic pressure and partially due to Ca^2+^ uptake with the activation of Ca^2+^-sensitive proteases. Additionally, the blockade of axonal transport and reduced nerve growth factor has also been implicated in the C-fibers defunctionalisation, thus ultimately leading to its antinociception [69,70].

Regarding the capsaicin sensitivity, previous reports have shown that the responsiveness to capsaicin varies among different species [71,72,73]. Cats and humans show well-established sensitivity to capsaicin, rabbits are less-sensitive when compared to rats and guinea pigs, whereas, birds and frogs are completely insensitive [55,74]. Treatment of adult rodent species (rat, mouse, and guinea pig) with doses in the range of 50–125 mg/kg of capsaicin resulted in the maximal degeneration of capsaicin-sensitive sensory neurons [75]. Whereas, treatment of neonatal rats with 50 mg/kg capsaicin lead to increased selectivity for unmyelinated afferent nerve fibers and small dark cell bodies in the sensory ganglia [76]. However, higher doses (>50 mg/kg) of capsaicin resulted in the ablation of few myelinated afferents and large light cell bodies [77]. Like mammals, birds also possess TRPV1 receptor, but birds TRPV1 remain insensitive to capsaicin compounds. In a previous study, Tewksbury et al., confirmed that seed-dispersing birds but not seed-destroying rodents consumed chili peppers. It was suggested that the rodents were deterred due to the presence of capsaicin compound in chili fruits [71]. Later, they supported their direct deterrence hypothesis by suggesting that fruiting plants produce fruits that attract seed dispersers and deter seed predators [72,73].

## 4. Capsaicin-Derived Agonists and Antagonists

In order to comprehend the actions of capsaicin, it is necessary to briefly introduce some capsaicin-derived agonists and antagonists. Resiniferatoxin (RTX) is regarded as an ultra-potent analogue of capsaicin that acts as an agonist of TRPV1 and displays several thousand-fold more potency than capsaicin. They both share the same vanillyl moiety that is essential for bioactivity. The structure of RTX has an intricate, rigid diterpene skeleton displaying a far more potent role than the simple hydrophobic core observed in capsaicinoids. RTX mimics the pharmacological actions of capsaicin in a highly potent manner [78]. Zucapsaicin, the *cis*-isomer of capsaicin, shows potent efficacy against episodic cluster migraine prophylaxis, episodic cluster headache, and alleviates neuropathic pain [79]. Capsazepine (CAPZ—a synthetic analog of capsaicin) derived by altering the chemical backbone of capsaicin is the first antagonist to be identified. Radio-ligand binding studies identified that CAPZ blocks the activation of TRPV1 by displacing RTX from its ligand-binding site [80]. Additionally, it also inhibits TRPM8, nicotinic, and voltage-gated Ca^2+^ channels. Even though CAPZ showed anti-hyperalgesic and anti-inflammatory activity in animal models of inflammatory and neuropathic pain, its efficacy varies among different species. Hence, CAPZ did not reach the clinical arena, but its derivatives are currently being investigated in several laboratories. The clinical status related to the therapeutic effects of other capsaicin-derived agonists and capsaicin-derived- and capsaicin-targeted antagonists are summarized in Table 1 and Table 2, respectively.

## 5. Pharmacological Actions of Capsaicin: Implications for Pain Control and More

The pungent principle capsaicin forms the most important pharmacological agent which can be easily obtained through our diet. Capsaicin is unique among naturally occurring irritants due to its ability to trigger a refractory state in the nerve terminal expressing TRPV1 as well as to generate prolonged nerve defunctionalisation at sufficient dosing [92]. The dual nature of capsaicin on sensory neurons (sensitization or desensitization) has been widely utilized as a pharmacological research tool to investigate the therapeutic intervention of capsaicin and its derivatives for the treatment of multiple disease states. The major part of capsaicin pharmacology focuses on treating several painful conditions. Additionally, the beneficial role of capsaicin has also been observed in the treatment of dermatological, gastrointestinal and cardiovascular conditions, various cancers, obesity, and several other pathologies [93].

### 5.1. Capsaicin-Based Therapies for Management of Chronic Pain

Chronic pain still represents a major treatment challenge for health-care providers due to its vague etiology, complex history, and poor responses to therapy. Chronic painful conditions include multiple forms of neuropathic pain, musculoskeletal pain, bone cancer pain, inflammatory bowel disease (IBD), and migraine [94,95]. Most often, capsaicin has been studied for PHN, HIV-DSP, musculoskeletal pain, and shingles. The search for the effective therapies against chronic pain is still ongoing. Capsaicin acts as a double-edged sword, displaying both pronociceptive and anti-nociceptive characteristics. Topical application of high-concentration capsaicin creams and gels have been utilized to treat several neuropathic pain conditions. Conversely, acute TRPV1 stimulation with capsaicin has been shown to induce pronociceptive effects resulting in the development of allodynia and hyperalgesia [96].

#### 5.1.1. Capsaicin and Neuropathic Pain

Neuropathic pain is described as the pain arising from lesions or disorder affecting peripheral or central sensory nerves, which is often characterized by tonic, unrelenting pain with sensation-related symptoms like burning, numbness, or hypersensitivity [92,97]. The pharmacological interest in capsaicin for neuropathic pain has resulted not only upon the identification of capsaicin receptor and its location on peripheral nerves, or its role in pain relief, but also due to its ability to produce degeneration of the nociceptor fibers upon repeated doses for overall long-term pain reduction. Local or topical application of capsaicin treatment has been in existence for a long period and much attention is being focused in this area of research. Topical capsaicin formulations are widely utilized for pain management. Capsaicin-based topical formulations have been studied using three varying concentrations (0.025% and 0.075%, regarded as “low dose” and 8%, regarded as “high dose”) in order to identify the most efficacious dose with less adverse effects [92].

Since the early 1980s, low-concentration capsaicin containing formulations, i.e., <1%, are being sold over-the-counter (OTC) in several countries for the treatment of neuropathic and musculoskeletal pain. The 2012 Cochrane review by Derre and Moore based on the existing data and literature information (seven studies involving a total of 1600 patients) assessed the potency of low-dose topical capsaicin in patients suffering from neuropathic pain [98]. The review drew the conclusion that low-concentration capsaicin does not seem to be particularly effective in the treatment of different forms of chronic neuropathic pain. While most of the available data suggest the ineffectiveness of low-concentration capsaicin in the management of musculoskeletal and multiple modalities of neuropathic pain, yet, a few studies reveal the topical application of low-concentration capsaicin as being of rational clinical benefit for the management of pain [92,99]. However, the shortcomings of low-concentration topical capsaicin including daily, multiple dosings at predetermined intervals associated with application-induced burning, possible contamination of the patient’s environment like bedding, lenses, clothing, etc., and its poor compliance have deterred unveiling its pharmacological significance [59].

In an attempt to overcome the limitations, formulations containing high-dose capsaicin were developed and evaluated. Robbins et al. primarily suggested the application of high-concentration topical capsaicin (5%–10%) for the handling of post-treatment pain [100]. The study included ten subjects suffering from intractable lower extremity pain with neuropathic characteristics. It was inferred that the single topical utility of high-concentration capsaicin under local anesthesia provided significant pain relief. Later, several reports showed the safety and efficacy of using 8% capsaicin patches in the management of neuropathic pain resulting from PHN, post-traumatic neuropathy, HIV-DSP, post-surgical neuralgia, and mixed pain syndrome. The 2013 Cochrane review evaluated the potency of high-dose capsaicin (8%) in patients suffering from PHN and HIV-DSP [101]. The review included six trials with a total of 2000 patients. The review inferred that topical use of high-dose capsaicin was more effective than “placebo” by rendering 30%–50% significant post-treatment pain reductions. Moreover, an advancement in the overall quality of life indicators like improved sleep pattern, decreased fatigue, and depression were noted. According to the Cochrane database, high-dose capsaicin maintained the efficacy, which lasted at both 8 and 12 weeks, whereas low-dose capsaicin applied multiple times daily over several weeks seemed less effective on neuropathic pain subjects [98,101]. Additionally, German pain centers demonstrated increased alleviation of pain in patients with localized neuropathic pain (PHN, HIV-DSP, back pain, and cervical spinal radiculopathy) by the utility of capsaicin 8% cutaneous patch [102].

Following positive trials of 8% capsaicin patch in PHN and HIV-DSP, EU approved its utility in both conditions in non-diabetic patients, while FDA approved its use only in PHN. Currently, capsaicin 8% topical patch is commercially available under the trade name, Qutenza (EU) or Transacin (U.S.) for the treatment of PHN. Phase I data suggested that a single, 60-min application was sufficiently enough to produce nociceptor defunctionalization [103,104]. Phase III data showed efficacy against PHN and HIV-DSP, where the efficacy lasted up to 12 weeks [104,105]. Recently, phase IV clinical trials were conducted to compare the efficacy of high-concentration capsaicin with standard therapy pregabalin in peripheral neuropathic pain (PNP). The capsaicin 8% patch rendered non-inferior pain relief to an optimized dose of pregabalin in PNP patients with a more rapid onset of action, fewer systemic adverse responses, and better treatment satisfaction [25]. Yet, high-concentration capsaicin therapy is not without its adverse effects including pain, pruritus, skin erythema, papules, and a transient increase in blood pressure [98,101]. To overcome this therapeutic barrier, a unique protocol for high-dose capsaicin administration is followed. Pretreatment of the application area with local anesthesia subsequent to the application of high-concentration capsaicin patch significantly reduced the burning and/or irritation adverse effects [106,107]. In general, the clinical outcomes of topical capsaicin in neuropathic pain conditions using both low- and high-doses have been discussed.

#### 5.1.2. Capsaicin and Musculoskeletal Pain

There are several conditions that can cause musculoskeletal pain including arthritic disorders, fibromyalgia, repetitive strain injury, backache, and other chronic muscle pain [108]. Mason et al. performed three double-blind placebo controlled trials on 368 patients for analysis of musculoskeletal pain with 0.025% low-dose capsaicin. The relative gain achieved with low-dose capsaicin was 1.5 compared to placebo and the number needed to treat was 8.1 [108]. The lower relative benefit of capsaicin 0.025% shows that it is less effective than topical NSAIDS in the management of chronic musculoskeletal pain.

Most clinical therapies for arthritis pain are directed orally, however, various topical formulations are also being utilized, including NSAIDS, capsaicin, and comfrey [109]. Low-dose topical capsaicin (0.025% or 0.075%) is commonly used to treat arthritis pain and sold OTC under the trade names Zostrix and Capzasin-P. Only a few studies have probed the use of topical capsaicin for treating arthritis, including osteoarthritis (OA), rheumatoid arthritis (RA), and fibromyalgia [99,110,111]. In a double-blind, randomized 4-week trial, 70 patients with OA and 31 patients with RA applied either capsaicin 0.025% cream or placebo to painful knees [110]. Most patients continued concurrent arthritis medications. After 4 weeks of capsaicin treatment, RA pain was reduced by 57% and OA pain was reduced by 33%. In another double-blind, placebo-controlled, randomized 4-week study, topical capsaicin 0.075% was studied for relieving hand pain in 21 patients with RA and OA [111]. Capsaicin was applied to each painful hand joint four times daily with an assessment at baseline, and then at 1, 2, and 4 weeks. Compared with placebo, capsaicin treatment decreased tenderness and pain linked with OA but not with RA. Casanueva et al. investigated the management of fibromyalgia using topical capsaicin 0.075% [99]. The study enrolled 126 patients, where they received their routine fibromyalgia treatment or usual regimen plus low-dose capsaicin. Results demonstrated a short-term improvement in patients with severe fibromyalgia upon addition of capsaicin to its routine treatment.

Several studies assessed the efficacy of capsaicin therapy in older patients suffering from OA of the hand [111], mixture of joints [112,113], and knee [110,114,115] for a period of 4–12 weeks. A comparison study involving 113 patients with OA pain, compared capsaicin 0.025% cream with placebo applied four times daily in a 12-week randomized trial [112]. At the end of the trial, 81% of patients using capsaicin vs. 54% using placebo were improved by physician’s global evaluation (*p* = 0.03). Patients treated with capsaicin reported increased pain reduction on the visual analog scale (VAS). Moreover, joint tenderness assessed by palpation decreased in the capsaicin group at week 4 (*p* = 0.03), week 8 (*p* = 0.01), and week 12 (*p* = 0.01) compared with placebo. Overall, capsaicin proved to be more efficacious than placebo in providing pain relief. Furthermore, another comparison study focused on the treatment of OA pain, where patients were applied either capsaicin cream 0.25% (2 times daily) or 0.025% (4 times daily) for 4-weeks [116]. This study showed that higher-concentration capsaicin offered increased pain relief and a more rapid onset of action relative to lower-concentration capsaicin. About one half of the patients utilizing 0.25% capsaicin twice daily presented at least a 50% reduction in pain severity after 2 days of treatment, whereas a 50% reduction in pain severity did not occur until day 14 in the patients receiving 0.025% capsaicin treatment.

McCleane et al. showed the efficacy of combined capsaicin therapy with glyceryl trinitrate in the management of painful OA [113]. About 200 patients with OA were topically applied one of the four creams: placebo, 0.025% capsaicin, 1.33% glyceryl trinitrate, or 0.025% capsaicin + 1.33% glyceryl trinitrate, over the affected joint for a period of more than 6 weeks. The study reported that topical capsaicin and glyceryl trinitrate portrayed an analgesic effect in painful OA. Upon combination, this effect seemed to be increased and being more tolerable when compared to capsaicin alone. Largely, analgesic consumption is reduced by capsaicin, glyceryl trinitrate and to a greater extent, by both combined. Additionally, another cross-over, double-blind, randomized controlled trial evaluated the efficacy of 0.0125% capsaicin gel compared to a placebo [115] in 100 knee OA patients. The results based on VAS and WOMAC scores highlighted that 0.0125% capsaicin gel can be considered as an effective treatment in treating mild to moderate painful OA knees. Schnitzer et al. showed the efficacy of civamide cream 0.075% over placebo (0.01% civamide) for the treatment of knee OA. A total of 695 patients were randomized to receive either civamide 0.075% or placebo for a period of 12 weeks to 1 year. This study showed that the efficacy of 0.075% civamide cream was well-tolerated compared to placebo [114]. The 2012 American College of Rheumatology guidelines for managing OA identify topical capsaicin as a conditional treatment for OA of the hand but do not recommend OA of the hip or knee [117]. An advantage of the utility of low-dose capsaicin in musculoskeletal pain is the absence of systemic adverse effects, which offers a treatment alternative for patients with intolerance or contraindications to conventional treatment. The most common adverse effect observed is transient burning sensation at the application site that rapidly decreases with continuing use. In summary, the beneficial effects of oral and topical application of low-concentration capsaicin in musculoskeletal disorders such as RA, fibromyalgia, and OA have been outlined.

#### 5.1.3. Capsaicin and Migraine

Chronic migraine is a general condition illustrated by a progressive increase in the frequency of attacks until the headache is permanently present, with frequent exacerbations [118,119]. Previous studies provided intranasal capsaicin as a new therapeutic option for the management of cluster headaches [120,121]. Later, two studies showed the efficacy of intranasal civamide for the management of migraine and episodic cluster headache [122,123]. Fusco et al. conducted a double-blind study to evaluate the effect of repeated doses of intranasal capsaicin on chronic migraineurs. Eight patients were enrolled; four were randomly allocated to capsaicin treatment and four to placebo. Capsaicin treated patients showed an improvement in their migraine, which was evaluated to be between 50% and 80%. Moreover, statistical evaluation (*p* < 0.01) portrayed that capsaicin was more effective than placebo [119]. Another study showed the efficacy of capsaicin jelly (0.1%) in reducing migraine in patients suffering from arterial pain at the onset of migraine [124]. Briefly, the acute treatment of migraine headache using intranasal civamide and capsaicin jelly have been summarized. 

### 5.2. Anti-cancer Effects of Capsaicin

Capsaicin has been reported to decrease cancer cell growth, cell cycle arrest, and trigger programmed cell death in several cancer cell lines, including adenocarcinoma, breast cancer, colon cancer, cutaneous cell carcinoma, esophageal carcinoma, gastric cancer, glioma, hepatocellular carcinoma, leukemia, multiple myeloma, nasopharyngeal carcinoma, non-small cell lung cancer, pancreatic cancer, prostate cancer, tongue cancer, and many others [125,126]. It has also shown to exhibit anticancer activity in vivo by decreasing the progression of several tumors in mice. Due to these roles, capsaicin has been considered as a new cancer therapy candidate. Previous studies showed that genotoxic and proapoptotic activities of capsaicin are rather restricted to cancer cells, whereas in normal cells, it fails to induce cell cytotoxicity [127,128,129,130]. The overall mechanism of capsaicin-triggered cancer cell apoptosis is not clearly understood. However, the intracellular events such as ROS generation, intracellular Ca^2+^ increase, activation of transcription factors (NF-κB and STATs), and disruption of mitochondrial membrane transition potential, as well as pathways including AMP-dependent kinase and autophagy in capsaicin-induced apoptosis, are well established [125]. The antitumor properties of capsaicin in various cancers and its underlying mechanisms have been well-documented in several other reports [93,125]. Only the recent studies that have been published in the year 2015 are mentioned in this section. A few previous studies on anticancer effects of capsaicin are summarized in Table 3.

Ferreira et al. performed both in vitro and in vivo studies of RPF151 (an alkylsulfonamide analogue of capsaicin) on MDA-MB-231 breast cancer cells and murine MDA-MB-231 model, respectively. In vitro study showed that RPF151 negatively regulated p21 and cyclins A, D1, and D3, resulting in S-phase arrest and apoptosis. Moreover, the in vivo anticancer activity of RPF151 was TRPV1-independent, thus suggesting its lower pungency compared to capsaicin [131]. A synergistic induction of apoptotic cell death and cell proliferation inhibition was observed in multiple colorectal cancer cell lines treated with the combination of capsaicin and 3,3’-diindolylmethane (DIM) through targeting transcriptional activation of NF-κB and p53 and activation of genes downstream of these transcription factors [132].

Apoptosis induction by eugenol and capsaicin in human gastric cancer cell line, AGS, was well-elucidated by the presence/absence of p53. In the presence of p53, capsaicin produced a more potent apoptotic induction than eugenol. However, eugenol was a better apoptotic compound than capsaicin in the absence of p53 [133]. Apoptosis triggered by capsaicin in hepatocellular cancer cell line, SMMC-7721 was investigated by Bu et al. The authors reported that capsaicin inhibited cell viability and triggered apoptosis with the production of ROS and constant disruption of the mitochondrial membrane transition potential. Additionally, capsaicin-induced JNK and p38 MAPK phosphorylations were observed [134].

Wang et al. studied the cytotoxic and antitumor characteristics of capsaicin in *K-ras*–transformed pancreatic cancer cells using both in vivo and in vitro analysis. Results showed that capsaicin inhibited NOX and induced severe ROS accumulation in *K-ras*–transformed cells compared with parental E6E7 cells. Moreover, capsaicin inhibited cell proliferation, prevented invasiveness of *K-ras*–transformed pancreatic cancer cells, and caused minimum toxicity to parental E6E7 cells. In vivo analysis demonstrated that capsaicin exhibited antitumor activity against pancreatic cancer and showed oxidative damage to the xenograft tumor cells [155]. Another study evaluated the effect of capsaicin in β-catenin signaling and a possible association with STAT3. The results suggest that capsaicin-triggered cell death in pancreatic cancer cells was linked to the inhibition of β-catenin signaling due to the dissociation of β-catenin / TCF-1 complex and the process was orchestrated by STAT3 [156]. Venier et al. showed the therapeutic effect of capsaicin as a radio-sensitizing agent in preclinical models of prostate cancer. Low-dose capsaicin differentially affects tumorous and normal epithelial cells through the inhibition of NF-κB activity in vitro. Furthermore, in vivo study revealed that oral consumption of capsaicin is well tolerated and extensively reduced the tumor growth rate, proliferative index, and NF-κB expression [157]. Another study by the same group investigated the chemopreventive effect of capsaicin on prostate cancer using TRAMP model. Chronic oral administration of capsaicin was shown to be safe, well-tolerated, and effective in reducing the metastatic burden of prostate cancer [158]. Interestingly, Zheng et al. reported that capsaicin negatively controlled the activity of androgen receptor at the mRNA and protein levels by restoring miR-449a profiling. Furthermore, augmented sensitivity of prostate cancer was observed by an increase in the expression of miR-449a in capsaicin treatment [159]. In a nutshell, the recent therapeutic effects of capsaicin in breast cancer, colorectal cancer, gastric cancer, hepatocellular carcinoma, pancreatic cancer, and prostate cancer have been reviewed.

### 5.3. Anti-Obesity Effects of Capsaicin

Due to the modern lifestyle, more and more people both from highly developed and developing countries suffer from obesity. Obesity is a chronic disease where a person has gained so much body fat that it might have a harmful impact on their health. When a person’s body weight is 20% greater than normal, then one is considered as obese. Obesity serves as a harbinger for several health issues, including heart disease, type II diabetes, certain types of cancer, obstructive sleep apnea, and OA [160]. One of the most promising areas of capsaicin research is its potential in combating obesity. In the recent decade, several laboratory and clinical studies have reported the therapeutic effect of capsaicin as an anti-obesity drug which will be discussed below.

The systematic review and meta-analysis by Whiting et al. suggests that both capsaicinoids and capsinoids could play a potent role in weight management along with exercise and dietary measures [161,162]. The authors also suggest that capsaicinoids could induce a synergistic effect on weight loss in combination with other bioactive ingredients [162,163]. Zhang et al. carried out long-term feeding experiments on wild-type (WT) and TRPV1 knockout (KO) mice. Upon consumption of high-fat diet, the oral consumption of capsaicin for 120 days successfully hampered the incidence of obesity in WT mice, but, not in TRPV1 KO mice. This shows that TRPV1 stimulation may decrease the number and size of fat cells, thereby preventing the development of adipogenesis and obesity [164].

Since obesity-induced diabetes is a hot research topic, Kang’s group studied the effect of capsaicin in such disease condition. The authors reported that dietary capsaicin could alleviate the metabolic dysregulation in KKAy (genetically obese diabetic) mice by increasing the production of adiponectin and its receptor. KKAy mice were fed with high-fat diet for 2 weeks, supplemented with 0.015% capsaicin for further 3 weeks and evaluated against non-supplemental controls. Dietary capsaicin reduced fasting glucose/insulin and triglyceride levels in the plasma and/or liver and production of inflammatory adipocytokine genes and macrophage infiltrations. Additionally, enhanced expression of adiponectin and its receptor in adipose tissue and/or plasma in parallel with the increase in the activation of fatty acid oxidation marker, hepatic adenosine monophosphate (AMP)-activated protein kinase were observed [165]. Another report regarding the obesity-induced inflammation and metabolic disorders was published by the same group. C57BL/6 obese mice received a high-fat diet for 10 weeks, supplemented with 0.015% capsaicin for another 10 weeks and compared with non-supplemental controls. Dietary capsaicin lowered fasting glucose, insulin, leptin levels, as well as decreased the levels of tumor necrosis factor-alpha (TNFα), monocyte chemo-attractant protein-1 (MCP-1), and interleukin (IL)-6 mRNAs in adipose tissue and liver. Remarkably, dietary capsaicin decreased obesity-induced glucose intolerance by increasing fatty acid oxidation in adipose tissue and liver [166].

Garami et al. suggested that intraperitoneal capsaicin (5 mg/kg) could desensitize the local afferent vagal nerve endings. Subsequent to desensitization, starving Wistar rats which were not fed for 120 hours tend to lose 20% more weight than the controls, suggesting defective hypometabolic adaptation [167]. Another group showed the impact of intraperitoneal capsaicin (10 mg/kg) plus a high-fat diet altering the levels of lipid-metabolism related proteins and thermogenesis in white adipose tissue [168]. Stearns et al. reported the effect of capsaicin on weight gain and fat content upon disruption of vagal signaling [169]. Male Sprague-Dawley rats were subjected to truncal vagotomy (selective vagal de-afferentation with capsaicin, or placebo). After feeding with high calorie diet for 11 months, the weights of vagotomized rats and de-afferented rats were 19% and 7% less than controls, respectively. It was evident that truncal vagotomy directed to the reduction in weight gain and visceral abdominal fat deposition, whereas, vagal de-afferentation, resulted in the decrease of visceral abdominal fat only.

Interestingly, there are several studies suggesting the significance of capsaicin and capsinoids in activating brown fat thermogenesis (BAT) and decreasing body fat in humans (refer for details in [170]). Capsaicin has also been reported to induce apoptosis and block lipid storage in 3T3-L1 preadipocytes and adipocytes [171]. Clinical studies supporting the theory of consuming capsaicin and its related food ingredients for lowering the prevalence of obesity are quite active. In a sequence of human studies, Yoshioka et al. reported that reduced food intake, diminished appetite, and increased diet-induced thermogenesis after consumption of red pepper were primarily due to the sensory or gastrointestinal effect of capsaicin [34,172,173,174]. Similarly, another group also assessed the relative sensory and gastrointestinal contribution to capsaicin-induced satiety and its effects on food ingestion [175]. Belza et al. subjected 80 obese patients on a hypocaloric diet for initial 4 weeks. Subsequently, the subjects who lost more than 4% body weight continued hypocaloric diet in addition to randomized supplement (capsaicin or other bioactive) or placebo for next 8 weeks. At the end of the trial, the supplement group lost 0.9 kg more than the placebo group without showing any systemic adverse effects [163]. Other clinical studies that support the role of capsaicin and its analogues in the management of obesity have been extensively reviewed elsewhere [170,176]. To summarize, the pharmacological actions of capsaicinoids and capsinoids as an anti-obesity drug have been outlined.

### 5.4. Role of Capsaicin in the Gastrointestinal System

Several researchers have carried out extensive studies with capsaicin in the area of gastroenterology (reviewed in detail elsewhere [177,178]). The gastrointestinal effects of capsaicin portray both beneficial and detrimental outcomes depending on the applied doses. Szolcsányi and Barthó were the first to report the double roles of capsaicin on peptic ulcer disease in rats [92]. The protective effects of capsaicin on gastric mucosa have been well documented in both animal and human studies. Red pepper was found to be more effective in decreasing the intensity of dyspeptic symptoms through desensitization of visceral nociceptive C-fibers [179]. Another study also supported the effects of repeated, long-term capsaicin ingestion in reducing the dyspeptic symptoms [180]. Capsaicin-induced gastroprotective effects against aspirin-induced gatroduodenal mucosal injury in humans were reported upon consumption of red pepper [181]. Moreover, a preliminary report showed that the long-term ingestion of red pepper in irritable bowel syndrome (IBS) patients with enteric-coated pills was considerably more effective in reducing the intensity of abdominal pain and bloating [182]. Dietary capsaicin also played a role in preventing non-alcoholic fatty acid disease via TRPV1-mediated peroxisome proliferator-activated receptor δ (PPAδ) [183]. In a prospective study of 84 healthy volunteers, the effects of low concentration capsaicin on the basal gastric acid output (BAO) and gastric transmucosal potential difference (GTPD), and ethanol- and indomethacin-induced gastric mucosal damage were tested [184]. Results showed that capsaicin inhibited the decrease in GTPD induced by ethanol and decreased the onset of micro-bleeding induced by indomethacin. The authors suggest that ingestion of capsaicin-containing foods reduced the incidence of gastric ulcers in patients consuming NSAIDS. Prakash and Srinivasan showed the beneficial effect of capsaicin in altering the brush border membrane permeability that is associated with augmented microvilli length and perimeter, ensuing increased absorptive surface of small intestine in rats [185]. Later, the same group demonstrated enhanced intestinal uptake of zinc absorption in rats fed on capsaicin [186]. Even though, capsaicin shows both beneficial and harmful clinical outcomes in gastroenterology, only its advantageous therapeutic effects have been discussed.

### 5.5. Role of Capsaicin in Cardiovascular Diseases

Previous studies have shown beneficial effects of dietary capsaicin on atherosclerosis, hypertension, cardiac hypertrophy, and stroke risk [187]. Daily intake of chili for 4 weeks has been reported to augment the resistance of serum lipoproteins to oxidation in adult men and women due to the antioxidant property of capsaicinoids [188]. The anti-aggregating effects of capsaicin on platelets via TRPV1-dependent or -independent mechanisms have also been studied [189,190,191,192]. It has been shown that capsaicin was able to pass through the plasma membrane of platelets and alter membrane fluidity [190]. Harper et al. showed that due to the presence of TRPV1 in human platelets, capsaicin provoked Ca^2+^ release from intracellular platelet stores and consequently contributed to ADP and thrombin induced platelet activation. LDL oxidation led to the development and progression of atherosclerosis. Capsaicin elicited the increase in the resistance of LDL to oxidation by delaying the initiation of oxidation and/or slowing the rate of oxidation [192]. Chronic activation of TRPV1 by capsaicin increased ATP-binding cassette transporter A1 (ABCA1) and reduced LDL-related protein 1 (LRP1) expression in aorta, thus attenuating atherosclerosis [193]. This shows that capsaicin is important in the prevention of cardiovascular disorders, including atherosclerosis and coronary heart disease. 

The cardiovascular system contains capsaicin-sensitive sensory nerves that help in the regulation of cardiovascular function through the release of calcitonin gene-related peptide (CGRP) via the stimulation of TRPV1 and SP [194,195]. The direct effects of capsaicin on the vasculature, relaxing basilar, meningeal, coronary, hepatic and mesenteric arteries of porcine and rodent have also been studied [196,197]. Yang et al. reported the positive effects of dietary capsaicin in lowering arterial blood pressure and improved endothelial function in genetically hypertensive rats via activation of endothelial TRPV1 and nitric oxide (NO)-dependent mechanism [198]. Another study demonstrated the effect of capsaicin in endothelium-dependent and -independent relaxation on rat mesenteric-artery which may be mediated by endothelial nitric oxide synthase (eNOS)-NO pathway and CGRP, respectively [199]. In stroke-prone spontaneously hypertensive rats, dietary capsaicin was found to provoke the activation and expression of eNOS, thereby leading to a delay in the onset of stroke [200]. A randomized clinical trial reported an increase in exercise time in patients with positive ischemic stress tests when transdermal capsaicin patches were utilized. NO levels were found to be augmented in the blood and this suggested that NO mediated arterial and venous vasorelaxation was a possible mechanism to elucidate this therapeutic effect [201]. To sum up, the role of dietary- and transdermal capsaicin in the treatment of cardiovascular diseases have been outlined. 

### 5.6. Role of Capsaicin in Dermatological Disorders

Several literature findings suggest a possible link between histamine-related itch and TRPV1. A previous study suggested that TRPV1-expressing neurons act as the main sensors and mediators of itch [202]. It was shown that histamine excited the TRPV1 expressed on sensory neurons via the activation of phospholipase A_2_ (PLA_2_) and lipooxygenase (LO). However, the definitive role of TRPV1 in histamine-mediated itch was well established in 2009, where it was shown that TRPV1-deficient mice displayed attenuated itch behavior in response to subcutaneous injection of histamine [203]. Topical capsaicin has been used in treating several dermatological disorders including alopecia areata, apocrine chromhidrosis, aquagenic pruritus, brachioradial pruritus, hemodialysis-associated pruritus, lichen simplex chronicus, lipodermatosclerosis, meralgia and notalgia paresthetica, PHN, ani, vulvae and scrota pruritus, prurigo nodularis, and psoriasis (refer to details in [204]). The antipruritic effects of topical capsaicin may be achieved via the defunctionalization of TRPV1-expressing primary afferents, induced by direct desensitization of the ion channel in the short term, and nerve terminal retraction mediated by excessive Ca^2+^ and blockade of mitochondrial respiration in the long term [59,205]. Moreover, immunohistochemical studies reported that the application of capsaicin mediates localized loss of nociceptive nerve fiber terminals in the epidermis and dermis [205,206]. Previous studies have shown that high-concentration, 8% transdermal capsaicin patch serve as a promising, useful adjuvant therapy in the treatment of PHN [104]. The effect of topical capsaicin in treating psoriasis vulgaris was studied using in situ hybridization. Hypoxia-inducible factor (HIF)-1α gene translation in psoriatic epidermis was found to be down-regulated upon capsaicin treatment for 21 days [207]. Pruritus is the most prevalent and distressing skin disorder in subjects receiving long-term hemodialysis. In such condition, pretreatment with capsaicin may help in alleviating hemodialysis-associated pruritus. In a placebo-controlled, double-blind, cross-over study of patients undergoing regular hemodialysis, topical application of 0.025% capsaicin relieved symptoms in 70% of patients compared to control group and the effect lasted up to 8 weeks post-treatment [208]. In another placebo-controlled study, topical 0.05% capsaicin showed significant anti-pruritic effect in patients receiving hemodialysis [209]. Topical 0.025% capsaicin was also demonstrated to exert moderate inhibitory effect on histamine, SP and proteinase-activated receptor (PAR)-2 agonist-induced itch responses [210]. Pruritus of the anogenital skin is characterized by intense itching and irritation in the genital or perianal area. In a placebo-controlled, randomized, crossover study, 31 of 49 patients experienced relief when treated with 0.006% capsaicin ointment compared to placebo menthol [211]. Lotti et al. studied the anti-pruritic effect of capsaicin cream using three different concentrations (0.025%, 0.5%, 1.0%) in patients with aquagenic pruritus [212]. The authors found that upon capsaicin application, the neuropeptidergic fibers were devoid of neuropeptides, which is responsible for mediating the disorder. Overall, the antipruritic role of capsaicin that shows a probable link between TRPV1 and histamine-related itch have been précised.

## 6. On-Target Adverse Effects of Capsaicin

With a rising interest in both food and pharmaceutical industries, the safety of capsaicin is an emerging source of concern for several researchers. Even though the benefits of capsaicin far outweigh its risks, it is necessary to delve into its therapy limitations. Capsaicin is considered as a powerful skin irritant even at low concentrations. This characteristic has been observed for generations among workers handling chili peppers who develop a contact dermatitis condition known as ‘Hunan hand’ due to prolonged exposure [213]. Capsaicin-containing products have been in clinical use for several years in treating several painful disorders. However, their effectiveness in analgesia is highly debated and some undesirable effects have been reported [17]. Capsaicin-induced dermal pain (stinging or burning) is common upon exposure to capsaicin-containing products [214]. Common adverse effects associated with the topical application of capsaicin seem to be small, transient, application-site reactions including pruritus, papules, and skin erythema [26]. Local, transient pain-related increases in arterial pressure during and immediately after the application procedure were also observed during clinical trials [215]. Moreover, coronary vasospasm and acute myocardial infarction were also documented upon the utility of topical capsaicin to relieve lower back pain [216]. These adverse responses can be quite unsought in subjects already experiencing pain thus limiting the utility of topical capsaicin in pain management [92]. Additionally, low-concentration capsaicin does not seem to be particularly effective in treating chronic musculoskeletal and neuropathic pain conditions [92]. Patient compliance with the capsaicin treatment can be difficult because nearly every patient undergo immediate cutaneous discomfort. Hence, several patients were not able to continue the therapy until the end of therapeutic benefit. Clinical studies showed that at least 30% of patients withhold from the capsaicin treatment due to lack of tolerability [217]. Furthermore, accidental inhalation of capsaicin could induce cough and mucosal burning [17]. The administration of capsaicin via topical or intradermal routes evoked severe chemogenic pain and produced an unstable area of dynamic mechanical allodynia [218]. Systemic application of capsaicin or its related compound elicited a pronounced fall in the body temperature due to a coordinated heat loss response accompanied by vasodilatation, salivation, and fall in metabolic rate at cool ambient temperature [67]. In contrast to the antitumor effects of capsaicin, it has also been shown to induce carcinogenesis in various animal, cellular, and human studies. Complete blockade of TRPV1 due to chronic exposure of capsaicin in the presence of a tumor promoter lead to increased skin carcinogenesis in mice [219]. Moreover, several epidemiologic reports suggest that consumption of hot chili peppers which contain varying amounts of capsaicin resulted in an increased risk of cancer, particularly, stomach, gall bladder, or gastric cancer (refer to details in [17,220]).

## 7. Conclusions and Future Perspectives

Capsaicin has elicited enormous interest for several centuries due to its conspicuous culinary and clinical applications. Despite its adverse effects, capsaicin is still being used as an active principle in several pharmaceutical formulations for treating various human ailments [16]. Moreover, emerging studies have shown that capsaicin is implicated in a broader range of functions than previously anticipated. Even though it is best characterized in the field of nociception and pain, several experimental and clinical studies also demonstrate its role in other important pathological states like cancer, obesity, skin disorders, cardiovascular diseases, etc. Additionally, it has also been implicated in other activities including treatment of the upper respiratory reflexes, prevention of adipogenesis, boosting metabolic rate, and regulation of innate and adaptive immune responses [221]. With the growing interest of the utility of high-dose capsaicin in painful neuropathic conditions, it has opened the gate for pharmaceutical sectors to propose a novel chapter of analgesics. Capsaicin 8% transdermal patch shows huge promise as an effective adjuvant therapy for the insistent pain of PHN. Topical capsaicin utilized as a single therapy or in conjunction with other analgesics offers a low-risk choice for patients who do not achieve control on other regimens and significantly improve their life quality. Moreover, several capsaicin analogues obtained naturally or synthetically guided in the identification of novel drugs with less or no lethal effects. In an attempt to minimize local side effects and increase the drug tolerability, several novel technologies have been introduced to improve capsaicin delivery [222,223,224,225]. The future challenges that remain to be addressed include determining the effective dose levels of capsaicin, and efficient mode of administration. Advanced SAR studies are needed to be designed for the identification of novel capsaicin analogues with less or no toxic effects. Furthermore, the continuing debate on whether the consumption or topical application of capsaicin is safe, need to be supported with more well-controlled epidemiological studies [17]. Despite these challenges, harnessing the therapeutic potential of capsaicin and its analogues will help in the development of new strategies for the treatment of a plethora of diseases.

## Figures and Tables

**Table 1:** Clinical status of capsaicin derivatives as agonists in diseases.

Compound *	Route	Clinical Indication	Clinical Status	References/ClinicalTrials.gov Identifier
NGX-4010 (Qutenza)	Topical	PHN	FDA approved	[81]
		PHN, HIV-DSP, and PNP	EMA approved	[24,25]
Zucapsaicin (Civamide)	Topical	PHN	Phase II	[79], NCT00845923
	Topical	Knee osteoarthritis	Phase III (completed)	NCT00995306, NCT00077935
	Spray	Dry eye syndrome	Phase II	NCT02116244
	Intranasal	Episodic cluster headache	Phase III (completed)	NCT00069082, NCT00033839
ALGRX-4975 (Adlea)	Direct instillation into the surgical site	Total knee arthroplasty	Phase III (completed)	[82], NCT00681356
	Injection	Postoperative pain	Phase II (completed)	NCT00133133, NCT00146198
	Injection	Morton’s neuroma	Phase II (completed)	NCT00130962
RTX	Intraganglionic/intrathecal injection and topical route	Morton’s neuroma, localized nerve injuries, burns, complex regional pain syndrome, amputation, corneal neuropathic, osteoarthritic, post-incisional, lower back, and chronic gynecological pain	Phase II (completed)	[83]
	Intrathecal injection	Cancer-induced bone pain	Phase 1b	NCT02522611
Nonivamide (Nicoboxil)	Topical	Acute lower back pain	Phase III (completed)	[84], NCT01708915
Capsiate	Oral	Antitumor, anti-inflammatory, analgesic, and weight management	Phase I (completed)	[85], NCT00692601

* Compounds’ trade names are mentioned in parentheses.

**Table 2:** Clinical status of capsaicin-derived- and capsaicin-targeted-antagonists in diseases.

Compound *	Route	Clinical Indication	Clinical Status	References/ClinicalTrials.gov Identifier
Capsazepine ^#^	Oral	Inflammatory, neuropathic pain (Guinea pigs)	Preclinical	[86]
A-425619 ^#^	Oral	Chronic inflammatory, post-operative, osteoarthritic, neuropathic pain	Preclinical	[87]
SB-705498 ^#^	Oral	Acute migraine, chronic cough, dental pain	Phase II (completed)	[88], NCT00269022, NCT01476098, NCT00281684
	Oral	Rectal pain	Phase II (terminated)	[88], NCT00461682
	Intranasal	Non-allergic- and allergic-rhinitis	Phase II (completed)	NCT01424514, NCT01424397
	Topical	Atopic dermatitis	Phase I (completed)	NCT01673529
ABT-102	Oral	Inflammatory, osteoarthritic, post-operative, and bone cancer pain	Phase I (completed)	[88,89], NCT00854659
AZD1386	Oral	Chronic pain	Phase I (completed)	NCT00736658
		Dental, esophageal pain	Phase II (completed)	[88], NCT01019928, NCT00672646
		Neuropathic, osteoarthritis pain	Phase II (terminated)	NCT00976534, NCT00878501
JNJ-39439335 (Mavatrep)	Oral	Pain, osteoarthritis	Phase I (completed)	NCT01006304, NCT01343303
PAC-14028	Topical	Skin pruritus, erythematotelangiectatic and papulopustular rosacea, atopic dermatitis	Phase II (completed)	NCT02052531, NCT02052999, NCT02583022
JTS-653	Oral	Overactive bladder pain	Phase II (discontinued)	[90]
		Post-herpetic neuralgia	Phase II (completed)	[91]
XEN-D0501	Oral	Chronic idiopathic cough, chronic obstructive pulmonary disease (COPD)	Phase II (completed)	NCT02233699, NCT02233686

* Compounds’ trade names are mentioned in parentheses. ^#^ Capsaicin derived antagonists.

**Table 3:** Summary of the antitumorigenic effects of capsaicin in several cancer cells/cell lines.

Cancer Type	Tumor Cells/Cell Lines Utilized	Major Outcomes	References
Breast Cancer	MCF-7, BT-20, SKBR-3, MDA-MB231, T47D, BT-474, MCF10A	Decreased mitochondrial membrane potential, cell-cycle arrest, apoptosis	[135,136,137,138,139]
Cholangiocarcinoma	TFK-1 and SZ-1	Modulation of Hedgehog pathway, apoptosis	[140]
Colon cancer	SW480, LoVo, HCT-116, CT-26, HT-29, CoLo320, Colo205	Cell cycle arrest, apoptosis, changes in cell morphology, DNA fragmentation	[141,142,143,144,145]
Gastric cancer	AGS, SNU-668, HGC-27	Apoptosis, inhibition of cell proliferation	[146,147,148,149]
Hepatocellular carcinoma	HepG2, Hep3B	Apoptosis	[150,151]
Pancreatic cancer	AsPC-1, BxPC-3, PANC-1	Apoptosis	[128,152]
Prostate cancer	LNCaP, PC-3, DU-145	Apoptosis, dissipation of mitochondrial inner transmembrane potential	[130,153,154]
